# Selective inhibition of CDK9 in triple negative breast cancer

**DOI:** 10.1038/s41388-023-02892-3

**Published:** 2023-11-24

**Authors:** Ebtihal H. Mustafa, Geraldine Laven-Law, Zoya Kikhtyak, Van Nguyen, Simak Ali, Alex A. Pace, Richard Iggo, Alemwork Kebede, Ben Noll, Shudong Wang, Jean M. Winter, Amy R. Dwyer, Wayne D. Tilley, Theresa E. Hickey

**Affiliations:** 1 Dame Roma Mitchell Cancer Research Laboratories, Adelaide Medical School, University of Adelaide, Adelaide, SA Australia; 2 Department of Surgery & Cancer, Imperial College London, London, UK; 3 Institut Bergonié, University of Bordeaux, Bordeaux, France; 4 Drug Discovery and Development, Clinical and Health Sciences, University of South Australia, Adelaide, SA Australia

**Keywords:** Breast cancer, Targeted therapies, Cancer models

## Abstract

Targeted therapy for triple-negative breast cancers (TNBC) remains a clinical challenge due to tumour heterogeneity. Since TNBC have key features of transcriptionally addicted cancers, targeting transcription via regulators such as cyclin-dependent kinase 9 (CDK9) has potential as a therapeutic strategy. Herein, we preclinically tested a new selective CDK9 inhibitor (CDDD11-8) in TNBC using cell line, patient-derived organoid, and patient-derived explant models. In vitro, CDDD11-8 dose-dependently inhibited proliferation (IC_50_ range: 281–734 nM), induced cell cycle arrest, and increased apoptosis of cell lines, which encompassed the three major molecular subtypes of TNBC. On target inhibition of CDK9 activity was demonstrated by reduced RNAPII phosphorylation at a CDK9 target peptide and down-regulation of the MYC and MCL1 oncogenes at the mRNA and protein levels in all cell line models. Drug induced RNAPII pausing was evident at gene promoters, with strongest pausing at MYC target genes. Growth of five distinct patient-derived organoid models was dose-dependently inhibited by CDDD11-8 (IC_50_ range: 272–771 nM), including three derived from *MYC* amplified, chemo-resistant TNBC metastatic lesions. Orally administered CDDD11-8 also inhibited growth of mammary intraductal TNBC xenograft tumours with no overt toxicity in vivo (mice) or ex vivo (human breast tissues). In conclusion, our studies indicate that CDK9 is a viable therapeutic target in TNBC and that CDDD11-8, a novel selective CDK9 inhibitor, has efficacy in TNBC without apparent toxicity to normal tissues.

## Introduction

Triple negative breast cancer (TNBC) accounts for approximately 10–15% of all cases and comprise a molecularly diverse group of tumours that lack positivity for the three major diagnostic and prognostic biomarkers clinically assessed in breast carcinomas: the estrogen receptor-α (ERα), the progesterone receptor (PR), and amplification or over-expression of the human epidermal growth factor receptor-2 (HER2) [1, 2]. Despite some promising molecular targets that have emerged from pre-clinical studies, no targeted therapy for TNBC has become standard-of-care. In part, this is attributed to the fact that TNBC are very heterogeneous in terms of actionable drivers, both among patients and among multi-clonal populations within a tumour [3–7]. Therefore, patients with TNBC remain clinically managed by cytotoxic chemotherapies in the adjuvant and neoadjuvant settings [8–10]. Among breast cancer subtypes, TNBC have a particularly aggressive disease course, with a high rate of local or systemic relapse within 5 years and disproportionately high mortality [11]. TNBC also occur more frequently in younger, pre-menopausal women [11]. These features highlight an unmet clinical imperative to discover and preclinically evaluate targeted therapies for TNBC.

Targeting cyclin dependent kinases (CDKs) is a strategy of burgeoning interest in the field of cancer therapeutics due to the diverse roles of CDK enzymes in regulating cell proliferation and transcription [12, 13]. CDKs are a family of serine/threonine kinases that interact with a regulatory cyclin protein to bind and subsequently phosphorylate a target substrate, usually resulting in activation of the target protein. Functionally, CDKs are categorized into two major groups: (1) Cell cycle CDKs (CDKs 1, −2, −4, −6) that control proliferation via orchestrated activation of cell cycle regulatory proteins and (2) Transcriptional CDKs (CDKs 7, −8, −9, −12, −13 and 19) that control cycles of mRNA transcription by regulating the activity of RNA Polymerase II (RNAPII) [14, 15]. Dysregulation of CDK expression or activity has been associated with tumorigenesis and progression of multiple cancers [13, 16], including breast cancer [17, 18]. Targeting transcriptional CDKs is a viable option for TNBC because they have key features of transcriptionally addicted tumours, including overexpression or amplification of the *MYC* oncogene [6, 19]. While targeting MYC in TNBC is an area of active research [20–22], targeting transcription provides a means to simultaneously target *MYC* as well as other oncogenic drivers of TNBC, thereby circumventing the problem of patient and tumour heterogeneity [23].

CDK9 is a rate-limiting regulator of RNAPII transcriptional activity that releases the polymerase complex from gene promoters to initiate elongation of mRNA transcripts [15, 24]. Short-lived mRNA transcripts, including *MYC*, are highly dependent upon CDK9 activity to sustain elevated expression. Reciprocally, MYC requires CDK9 to function as a transcription factor that amplifies transcription to drive tumour growth [23, 25]. TNBC express CDK9 and therapeutic inhibition has been proposed as candidate target strategy [26–28], but pursuing this approach clinically has been limited by the lack of potent, highly specific CDK9 inhibitors. Although several are in phase I clinical trials for haematological or solid cancers, off target toxicity and poor oral bioavailability has been problematic, indicating a need for more selective drugs with better pharmacodynamic properties [23, 29].

As part of a medicinal chemistry and drug discovery program, we identified CDDD11-8 as a potent, orally bioavailable CDK9 inhibitor selective for CDK9 (with >50-fold selectivity over other CDKs assessed) that reduced in vitro and in vivo growth of human cell line models of acute myeloid leukaemia [30]. Herein, we tested the pre-clinical efficacy of CDDD11-8 in diverse human models of TNBC, including patient-derived organoids and explant cultures, and performed mechanistic analyses to support the strategy of CKD9 inhibition as a targeted therapeutic approach to treat women with this highly aggressive disease.

### Plasmid availability

The reporter plasmids engineered for this study (pDRM18 “LTN”, pDRM98 “KB”, pDRM166 “LKB”, pDRM209 “LTP”, and pDRM210 “LGP”), along with maps and sequences, have been deposited with Addgene (IDs #174721, 174720, 183502, 174723, and 174722, respectively). Plasmids are available for non-commercial use.

## Results

### Therapeutic efficacy of CDDD11-8 in TNBC cell line models

**Fig. 1:**
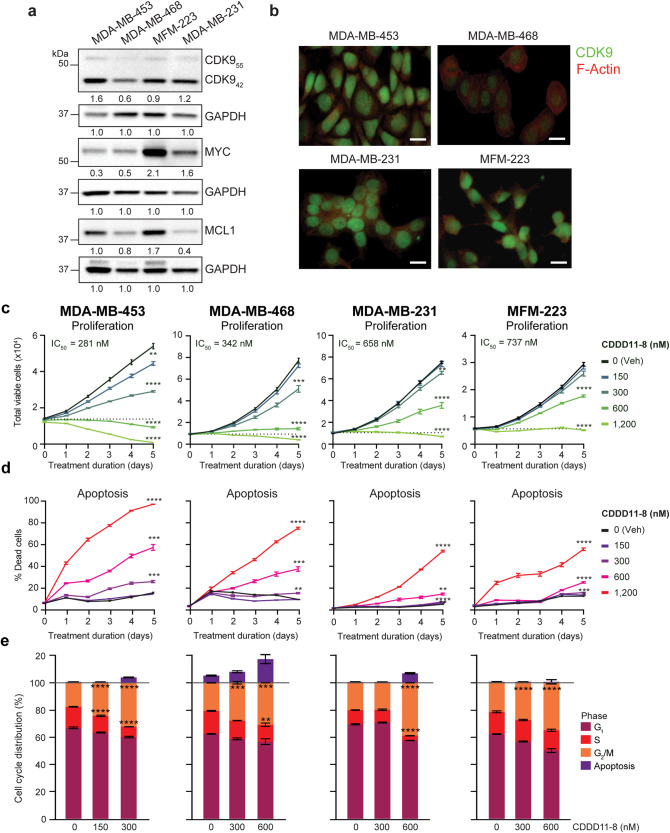
CDDD11-8 inhibits proliferation, promotes cell cycle arrest, and increases apoptosis of TNBC cell lines in vitro. **a** Representative Western Blots showing basal expression of CDK9, MYC, MCL1 and GAPDH (loading control) in four TNBC cell lines. Relative densitometry levels of protein (under the blot) are normalized to GAPDH levels on the same blot, set to a value of 1. **b** Representative dual-label immunofluorescence images of CDK9 (green) and F-Actin (red) in TNBC cell lines. Scale bar = 20 µm. **c** Representative growth curves showing proliferation of TNBC cell lines in response to escalating doses of CDDD11-8. Each data point represents an average of 4 images taken over 24 h. Data was compared using a two-way repeated measures ANOVA (Interaction: *F* = 712.5, 333.1, 333.0, and 326.3 for MDA-MB-453, MDA-MB-468, MDA-MB-231, and MFM-223 cells, respectively; d.f. = 40 and *p* < 0.0001 for each test). Asterisks denote a statistically significant difference compared to vehicle at endpoint, as determined by Dunnett’s multiple comparisons test. IC_50_ values were derived as an average of three independent proliferation assays for each cell line (shown in Supplementary Fig. 1a). **d** Death curves that correspond to growth curves in (**c**) in which apoptosis was determined by the ratio of Caspase-3/7 positive cells to live cells. Data was compared using a two-way repeated measures ANOVA (Interaction: *F* = 320.7, 213.1, 563.3, and 141.4 for MDA-MB-453, MDA-MB-468, MDA-MB-231, and MFM-223 cells, respectively; d.f. = 40 and *p* < 0.0001 for each test). Asterisks denote a statistically significant difference compared to vehicle at endpoint, as determined by Dunnett’s multiple comparisons test. Data shown in (**c**) and (**d**) represent the mean ± S.E.M. of 5 technical replicates per condition. **e** Stacked bar plots showing changes in cell cycle distribution in four TNBC cell line models after treatment with CDDD11-8 (MDA-MB-453 cells: 300 nM; MDA-MB-468, MDA-MB-231, MFM-223 cells: 600 nM). MDA-MB-453, MDA-MB-468 and MFM-223 were treated for 3 d, MDA-MB-231 for 5 d. Data reflects the mean ± S.E.M. of three technical replicates per condition. Cell cycle and CDDD11-8 concentration were compared for each cell line using a two-way ANOVA (Interaction results: MDA-MB-453 *F* = 181.6, MDA-MB-468 *F* = 22.79, MDA-MB-231 *F* = 542.8, MFM-223 *F* = 46.48; d.f. = 6 and *p* < 0.0001 for all tests). Comparison of cell cycle stages was performed based on total living cells, set at 100%. The fraction of dead cells in sub-G_1_ represented above the live cell analysis is based on total cells detected in the FACS assay. Asterisks denote significant difference between G2/M and S cell cycle phases compared to vehicle, as determined using Dunnett’s multiple comparisons test. *p < 0.05, **p < 0.01, ***p < 0.001, ****p < 0.0001.

To test the effect of CDDD11-8 on cell proliferation and viability, we investigated a panel of four cell lines that represent three major molecular subgroups of TNBC as described:[31, 32] Basal-like 1 (MDA-MB-468), Mesenchymal-like (MDA-MB-231) and Luminal-like (MDA-MB-453, MFM-223). Two CDK9 protein isoforms were detected; a major 42 kDa protein (CDK9_42_) and a minor 55 kDa protein (CDK9_55_) (Fig. 1a), consistent with CDK9 expression in mammalian tissues [33, 34]. The dominant CDK9_42_ isoform hereafter is referred to as CDK9. By Western Blot analysis, MDA-MB-453 cells had the highest and MDA-MB-468 cells the lowest level of CDK9 expression relative to the corresponding GAPDH protein loading control (Fig. 1a). Assessment of CDK9 expression by immunofluorescence supports much lower expression in the MDA-MB-468 cells compared to the other three cell lines that had a similar, higher level of expression (Fig. 1b). Baseline protein levels of key oncogenic CDK9 gene targets, MYC and MCL1, varied among the TNBC cell lines, but were generally higher in the MFM-223 model (Fig. 1a). Hence, this panel of TNBC cell line models provided a diversity of molecular subtypes and baseline expression levels of key targets with which to investigate the efficacy of our new CDK9 inhibitor.

The four TNBC cell line models were treated with twofold increasing concentrations of CDDD11-8 (150, 300, 600 and 1200 nM) and nuclei counts assessed via live imaging over a period of five days. Treatment with the CDK9 inhibitor significantly reduced cell proliferation in a time and dose-dependent manner in all models, with varying degrees of sensitivity (Fig. 1c; Supplementary Fig. 1a). The MDA-MB-453 cell line was most sensitive to CDK9 inhibition with an IC_50_ (derived from 3 independent experiments) of 281 nM. The MDA-MB-468 cell line was the second most sensitive (IC_50_ = 342 nM), followed by MDA-MB-231 (IC_50_ = 658 nM) and MFM-223 (IC_50_ = 737 nM). The observed anti-proliferative effects were accompanied by apoptotic cell death in a dose and time-dependent manner (Fig. 1d). As CDDD11-8 induced significant growth inhibitory effects (Fig. 1c), a dead/live cell ratio was calculated in the assessment of apoptosis to account for fewer cells at higher drug doses. Apoptosis was more substantial in MDA-MB-453 and MDA-MB-468 cells compared to MDA-MB-231 and MFM-223 cells. Consistent with these results, treatment with the CDK9 inhibitor induced G2/M cell cycle arrest across models (Fig. 1e, Supplementary Fig. 1b). The percentage of live cells undergoing S phase was reduced in all cell lines except for the MFM-223 line, the least sensitive model (Fig. 1e). Collectively, these results provide evidence that CDDD11-8 has growth inhibitory effects through both apoptosis and G2/M cell cycle arrest. Interrogation of the Dependency Map (DepMap) portal (www.depmap.org) revealed CRISPR gene scores for CDK9 below −1.0, indicating CDK9 is an essential gene in all of the cell line models of TNBC (Supplementary Fig. 1c). The RNAi scores for CDK9 knockdown indicated partial suppression and aligned with our CDDD11-8 results, whereby the sensitivity hierarchy was MDA-MB-453 > MDA-MB-468 > MDA-MB-231 > MFM223 (Supplementary Fig. 1c). This public data supports CDK9 activity as a vulnerability in TNBC and the concept that CDDD11-8 inhibits cancer cell fitness by targeting CDK9.

### CDDD11-8 reduces Ser2 phosphorylation of RNAPII and expression of target oncogenes

**Fig. 2:**
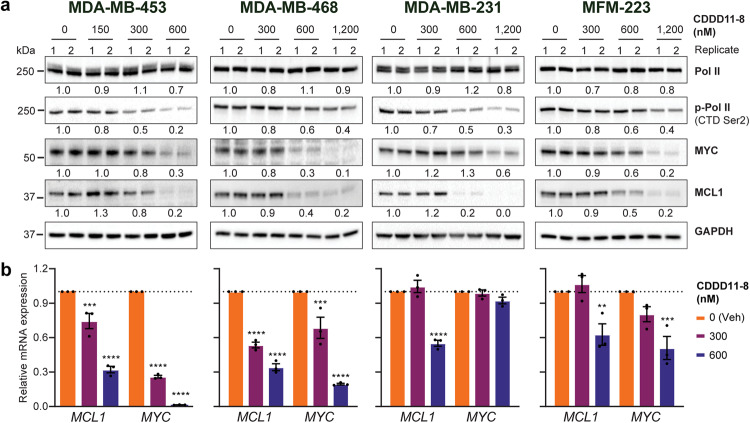
CDDD11-8 reduces expression of CDK9 targets. **a** Representative immunoblots showing the effect of CDDD11-8 on RNA polymerase II (Pol II), phosphorylated RNAP II at Serine 2 in the C-terminal domain (p-Pol II CTD Ser2), MYC, and MCL1 protein expression in TNBC cell lines after 6 h of treatment. GAPDH was used as a loading control. The average densitometry is normalized to GAPDH and presented as relative to vehicle treated cells. **b** Bar graph of RT‐PCR data for expression of CDK9 target genes *MYC* and *MCL1* after 4 h treatment with CDDD11-8. RT-PCR data was normalized to GAPDH and presented as relative to vehicle. Data represents the mean ± S.E.M. of 3 technical replicates per condition. Data was analysed using a two-way ANOVA (CDDD11-8 concentration *F* = 394, 160.4, 57.84, and 22.76 for MDA-MB-453, MDA-MB-468, MDA-MB-231, and MFM-223 cells, respectively; d.f. = 2, *p* < 0.0001 for each test). Asterisks denote a significant difference between treatment and vehicle, as determined using Dunnett’s multiple comparisons test. *p < 0.05; **p < 0.01; ***p < 0.001; ****p < 0.0001.

We next examined expression of CDK9 downstream targets, including phosphorylated Ser2 on the RNAPII C-terminal domain and two oncogenes (MYC, MCL1) in TNBC cells following treatment with CDDD11-8 (300 and 600 nM) for 4 h (gene expression) or 6 h (protein analyses). The drug significantly reduced Ser2 phosphorylation on RNAPII in a dose-dependent manner across all TNBC cell lines, without affecting total RNAPII levels (Fig. 2a), indicative of CDK9 inhibition. In MDA-MB-453 cells, the most sensitive model, phosphorylation of Ser2 on RNAPII was reduced by approximately 80% after a 6 h treatment with 600 nM of drug (Fig. 2a). This effect was accompanied by >70% reduction in the expression of MYC and MCL1 at the mRNA and protein level (Fig. 2a, b). RNAPII Ser2 phosphorylation was inhibited by approximately 40% in both MDA-MB-468 and MFM-223 cells after treatment with 600 nM CDDD11-8 (Fig. 2a). This inhibition coincided with a significant reduction in MYC and MCL1 mRNA and protein (Fig. 2a, b). In MDA-MB-231 cells, MCL1 expression was nearly ablated by 600 nM of drug (Fig. 2a), but a higher dose of 1,200 nM was required to decrease MYC protein expression (Fig. 2b). Together, these data are consistent with CDDD11-8 specifically targeting CDK9 activity via modifications of RNAPII that result in reduced expression of short-lived oncogenic transcripts in TNBC cells, which mechanistically accord with the drug’s inhibitory effects on cell proliferation and viability.

### CDDD11-8 induces RNAPII promoter pausing at G2/M checkpoint and MYC target genes

**Fig. 3:**
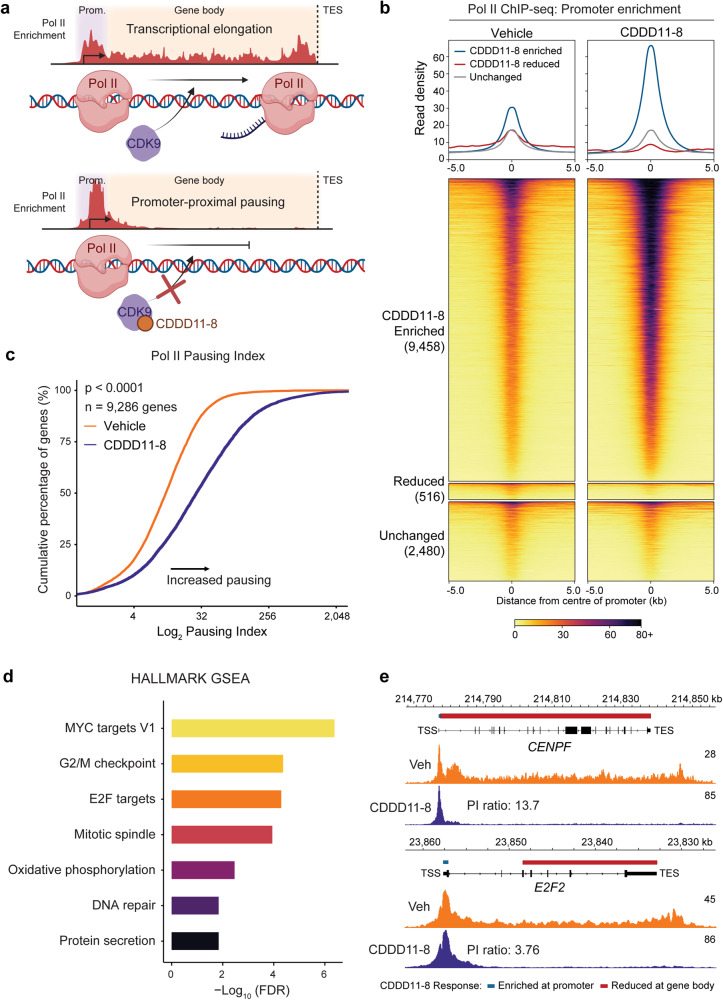
CDDD11-8 induces promoter-proximal pausing in MDA-MB-453 cells. **a** Schematic showing RNA polymerase II (Pol II) localization and corresponding Pol II enrichment by ChIP-seq over promoters and gene bodies during normal transcriptional elongation (upper panel) and during promoter-proximal pausing (lower panel). **b** Average read density plots (top panels) and heatmaps (bottom panels) representing Pol II ChIP-seq data, showing increased enrichment of Pol II at gene promoters after 4 h treatment with CDDD11-8 (600 nM) in MDA-MB-453 cells. Heatmaps are broken into three regions (increased, decreased, and no change with CDDD11-8), determined by differential enrichment analysis using FDR < 0.05. Data is presented as an average of two replicates representing independent passages of cells. **c** Average empirical cumulative distribution function plot showing a rightward shift in Pol II pausing index at promoters after treatment with CDDD11-8 (as defined in (**b**)) in MDA-MB-453 cells. The pausing index was defined as a log-transformation of the ratio between the Pol II promoter density and gene body density, where promoters were defined between – 50 bp and + 300 bp of the TSS, and gene bodies defined between – 300 bp and + 3 kb of the TES. Data is presented as an average of two replicates representing independent passages of cells and analyzed using a paired two-sided Wilcoxon test (W = 39,578,209, *n* = 9,286, *p* < 0.0001). **d** Gene ontology analysis for Pol II ChIP-seq data at CDDD11-8 enriched promoters identified in (**b**), compared to promoter regions that were not differentially gained with treatment. Over-represented HALLMARK gene sets and their corresponding log-transformed FDR values are shown for gene sets which met an FDR-corrected *p* value (q) of <0.05. **e** Representative genome browser tracks showing the average Pol II ChIP-seq signal in MDA-MB-453 cells at select HALLMARK G2/M checkpoint binding sites where Pol II promoter-proximal pausing was induced by CDDD11-8. Tracks are scaled to the maximal value (as indicated numerically at the upper-right of each track) to highlight changes in Pol II enrichment over the gene body. The pausing index (PI) ratio between CDDD11-8 and vehicle-treated cells are inset into the CDDD11-8 Pol II tracks. Data is presented as an average of two replicates representing independent passages of cells. The genomic tracks for *E2F2* have been horizontally scaled for ease of visualization, as they are normally oriented on the minus strand.

To examine the functional consequences of CDDD11-8 treatment on RNAPII chromatin distribution and promoter-proximal pausing, which is regulated by CDK9 (Fig. 3a), chromatin immunoprecipitation (ChIP)-seq analysis was performed on our most sensitive model. MDA-MB-453 cells were treated for 4 h with either vehicle or CDDD11-8 (600 nM). RNAPII ChIP-seq data generated from independent replicate experiments was highly concordant (Supplementary Fig. 2a). Global RNAPII enrichment over promoters increased approximately twofold with CDDD11-8 treatment (Supplementary Fig. 2b). Differential enrichment analysis revealed that treatment with the CDK9 inhibitor significantly increased RNAPII enrichment at >75% of promoters (Fig. 3b, Supplementary Fig. 2c). We then calculated the RNAPII Pausing Index at each gene associated with differential RNAPII promoter enrichment. CDDD11-8 treatment caused widespread RNAPII pausing (*p* < 0.0001), whereby 63% of assessed genes had >2-fold more paused RNAPII compared to elongating RNAPII (Fig. 3c, Supplementary Table 1). Over-represented in these paused promoters were MYC target, E2F target, and G2/M checkpoint genes (Fig. 3d, e, Supplementary Fig. 2d). Motif analysis of the paused promotors revealed enrichment of E2F DNA binding sites in addition to motifs for other transcription factors implicated in the pathology of TNBC (e.g., MYB, YYI [35, 36]) (Supplementary Table 1). These data accord with our cell cycle and molecular analyses, and support the specificity of CDDD11-8 for CDK9 through inhibition of RNAPII-mediated elongation of mRNA transcripts.

### CDDD11-8 inhibits in vivo growth of mammary intraductal TNBC xenografts

**Fig. 4:**
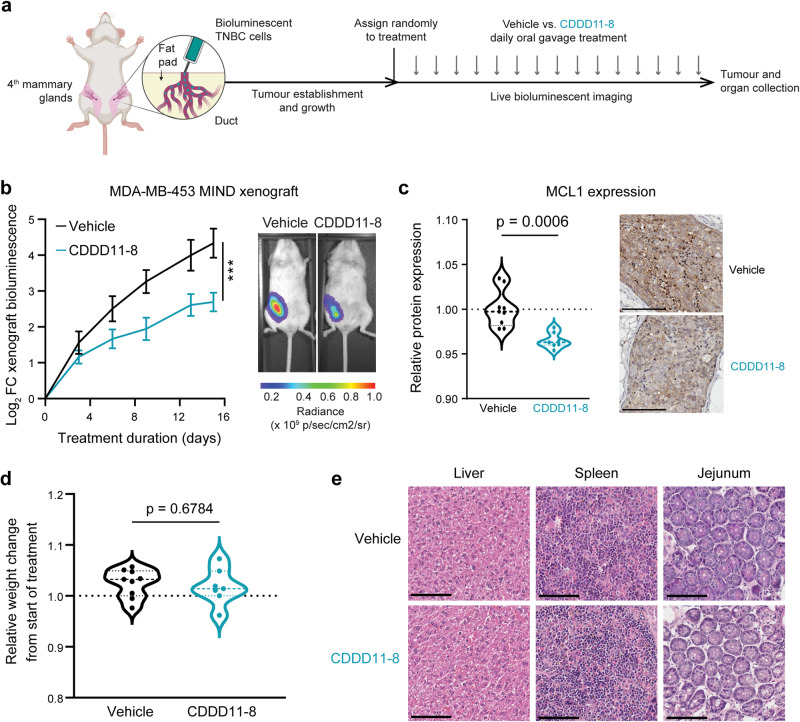
CDDD11-8 inhibits growth of TNBC mammary intraductal xenograft tumours. **a** Schematic of the Mammary Intraductal (MIND) xenograft model. Mice were inoculated with TNBC cells 5 d prior to beginning therapeutic treatment with either vehicle or CDDD11-8 (150 mg/kg/day). Treatments were administered daily (arrows). **b** Left panel: Tumour growth curves assessed using in vivo bioluminescence of MDA-MB-453 cells expressing luciferase. Data was analysed using a two-way repeated measures ANOVA (Interaction: *F* = 5.098, d.f. = 5, *p* = 0.0005), and least squares regression (Vehicle: R^2^ = 0.6890, *k* = 0.3988; CDDD11-8: R^2^ = 0.6599, *k* = 0.3708). Right panel: Representative bioluminescent images of mice with MDA-MB-453 MIND xenograft tumours at endpoint after treatment with vehicle or CDDD11-8. **c** Quantification and representative immunohistochemical images of MCL1 expression in MDA-MB-453 MIND xenografts. Data was analysed using an unpaired Welch’s test (*t* = 4.648, d.f. = 11.42, *p* = 0.0006). Scale bars represent 250 µm. **d** End-point bodyweights of adult NSG mice used for MDA-MB-453 MIND xenograft studies (Welch’s *p* = 0.6784). Data are presented relative to entry body weight. **e** Representative H&E staining images of mouse organs after 16 days daily treatment with either a vehicle or CDDD11-8. Scale bars = 50 µm.

To examine the in vivo efficacy of CDDD11-8, we first established mammary intraductal (MIND) xenografts using the MDA-MB-453 cell line, which was most sensitive to the drug in vitro. This MIND xenografting methodology recapitulates the ductal environment in which breast cancers normally arise [37, 38], so is considered more clinically relevant than conventional mammary fat pad xenografting. Adult female NSG mice (*n* = 20) were injected unilaterally with MDA-MB-453 cells expressing luciferase (Fig. 4a). After an intitial engraftment period of 5 d, mice were allocated by simple randomisation to treatment groups. The administered dose of CDDD11-8 was based on the maximal tolerated in vivo dose of CDKI-73, a first-generation, less specific CDK9 inhibitor [39]. NSG mice received either vehicle (0.1 M sodium acetate, pH 4.5) or CDDD11-8 (150 mg/kg/day) for 15 days by oral gavage (Fig. 4a). The drug significantly reduced growth of MDA-MB-453 MIND xenograft tumours, as determined using bioluminescent imaging (Fig. 4b) and reduced protein expression of MCL-1 in tumours (Fig. 4c; Supplementary Fig. 3a). No apparent behavioural or body weight changes were observed in treated mice (Fig. 4d, *p* = 0.6784). There was also no effect of CDDD11-8 on liver, spleen and intestinal histology (Fig. 4e), indicating absence of toxicity at this dose and period of time. A second in vivo experiment was performed using the MDA-MB-468 model under a similar experimental design, with the exception that tumours were innoculated bi-laterally and a higher dose of drug (200 mg/kg/day) was administered based on results of our recent study characterizing the pharmacokinetics and toxicity of CDDD11-8 in nude mice [30]. The second in vivo experiment also demonstrated drug-mediated growth inhibition (Supplementary Fig. 3b) with no associated animal toxicity (Supplementary Fig. 3c), including lack of effect on proliferative capacity of intestinal cells (Supplementary Fig. 3d) and number of neutrophils present in the spleen (Supplementary Fig. 3e). Collectively, these data show that CDDD11-8 is non-toxic in NSG mice at the tested doses and has modest but statistically significant in vivo growth inhibitory effects in two molecularly distinct cell line models of TNBC.

### CDDD11-8 had no effect on the histology or proliferative index of normal human breast tissues

We next examined the effect of CDDD11-8 on human tissue using patient-derived explants (PDEs; *n* = 4 independent cases) of histologically normal, non-malignant human breast tissues obtained from women undergoing reduction mammoplasty surgery and cultured ex vivo on a gelatine sponge scaffold (Supplementary Fig. 4a). This pre-clinical model sustains tissue architecture, viability, cellular complexity, and hormone responsiveness of breast tissue [40], and is thereby more clinically relevant than the common practice of testing normal tissue toxicity using the MCF-10A breast epithelial cell line. The MCF-10A cell line has some features of normal breast epithelial cells but was derived from a benign proliferative lesion, is a spontaneously immortal cell line with high basal proliferative capacity in 2D culture and has a phenotype under 3D culture that is not representative of normal human breast tissue [41, 42]. Breast PDEs were treated with vehicle or 3-fold increasing doses (300, 900 & 2700 nM) of CDDD11-8 for 48 h and harvested for assessment of histological features and proliferative index (% Ki-67 positivity). Compared to vehicle-treated explants, CDDD11-8 did not affect the histology or proliferative index of normal breast epithelial cells at any dose (Supplementary Fig. 4b, c). The absence of toxicity to CDDD11-8 in normal mouse tissues and human breast tissues is consistent with a study showing that phosphorylation of the CTD of RNAPII is not required for basal transcription [43], indicating that inhibition of CDK9 kinase activity should not be toxic to normal cells whereas transcriptionally addicted cancers are dependent on this activity [19].

### CDDD11-8 inhibits growth of patient-derived breast organoid models

**Fig. 5:**
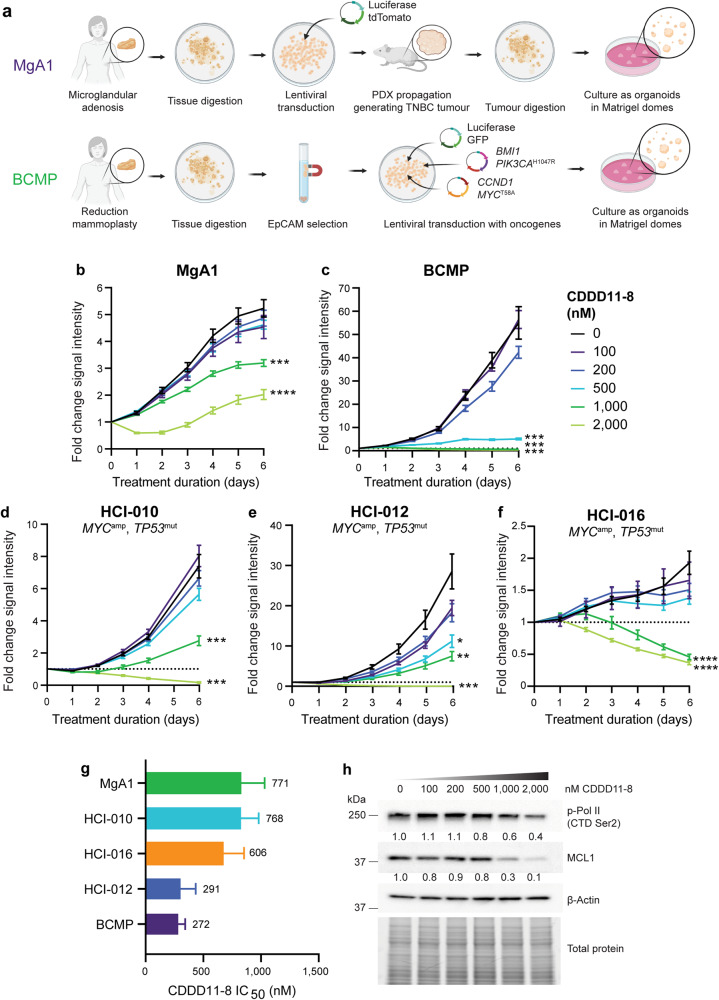
CDDD11-8 inhibits proliferation of patient-derived breast organoid models. **a** Schematic of the two patient-derived breast organoid models (MgA1, BCMP) generated for use in this study. Representative growth curves showing (**b**) MgA1, (**c**) BCMP, and two previously described patient-derived breast cancer organoid models (**d**) HCI-010, (**e**) HCI-012, and (**f**) HCI-016 proliferation in response to escalating doses of CDDD11-8 (MgA1 *F* = 18.44, d.f. = 30, *p* < 0.0001; BCMP *F* = 57.65, d.f. = 30, *p* < 0.0001; HCI-010 *F* = 36.24, d.f. = 25, *p* < 0.0001; HCI-012 *F* = 20.57, d.f. = 30, *p* < 0.0001; HCI-016 *F* = 5.67, d.f. = 30, *p* < 0.0001). Graphed data in (**b–f**) represents the mean ± S.E.M. of 8–10 replicate wells per condition. Independent experiments were performed at least twice. Asterisks in (**b–f**) denote a statistically significant difference compared to vehicle at endpoint, as determined by Dunnett’s multiple comparisons test. **f** Bar graph showing the average endpoint CDDD11-8 IC_50_ values for MgA1, HCI-010, HCI-016, HCI-012, and BCMP based on data presented in (**b–f**). IC_50_ values were determined based on the percent of total response and fit using a four-parameter logistic function, restrained between 0 & 100%. Error bars correspond to the 95% confidence interval. **h** Representative immunoblot data showing dose-dependent changes in phosphorylated RNA polymerase II (p-Pol II CTD Ser2) and MCL1 protein expression in BCMP organoids after 6 h treatment with CDDD11-8. Β-Actin and total protein content are provided as loading controls. Densitometry results are presented below each set of bands, normalized to total protein content, and presented as relative to vehicle (0 nM CDDD11-8) treated cells.

Owing to their 3D architecture and greater cellular complexity compared to traditional 2D cell line models, patient-derived organoid (PDO; PDxO) models are considered valuable pre-clinical tools for evaluation of targeted therapies for breast cancer [44]. First, therapeutic efficacy of CDDD11-8 was evaluated using two novel breast PDO models. The MgA1 model was derived from a small TNBC tumour that was surrounded in the patient by pre-malignant microglandular adenosis and established as a PDX by grafting into the mammary ducts of immunocompromised mice (Supplementary Fig. 5a). In contrast, the BCMP model was created from normal human breast epithelial cells isolated from a reduction mammoplasty and genetically engineered to overexpress four oncogenes: BMI1, CCND1, MYC^T58A^, and PIK3CA^H1047R^ (Fig. 5a). PDOs representing each model were treated with increasing doses of CDDD11-8 and changes in growth monitored using live cell imaging. As shown by comparisons with vehicle-treated controls, CDDD11-8 significantly inhibited growth of MgA1 and BCMP organoids in a concentration- and time-dependent manner (Fig. 5b, c). In contrast, organoids developed from the unmodified breast epithelial cells derived from two independent reduction mammoplasty samples were comparatively resistant to the CDK9 inhibitor (Supplementary Fig. 4d). The efficacy of CDDD11-8 was next tested in two previously described PDxO models (HCI-010, HCI-012) generated from PDXs of advanced, metastatic breast cancer established from women with chemotherapy-resistant disease, and one (HCI-016) generated from a PDX established from a metastatic lesion of unknown treatment history [44]. The HCI-010 and HCI-016 models are classified by the PAM50 gene signature as basal-like TNBC while the HCI-012 model is HER2-enriched; all three PDxO models had amplification of the *MYC* oncogene and inactivating mutations in the *TP53* tumour suppressor gene, the most common genomic abnormalities in TNBC. Treatment with CDDD11-8 dose-dependently inhibited growth of these organoids (Fig. 5d–f), with evidence for regression at a 2 µM dose. This data further supports the efficacy of CDK9 inhibition in advanced ERα-negative, PR-negative breast cancers, including TNBC and HER2-enriched contexts. Collectively, the five models resulted in endpoint IC_50_ values ranging from 272 to 771 nM (Fig. 5g). The BCMP model was the most sensitive to CDK9 inhibition (Fig. 5c, g), likely due to the exceptionally high rate of proliferation driven by ectopic overexpression of four oncogenes, supporting the importance of CDK9 in sustaining the high rate of transcription characteristic of rapidly proliferating tumours. Dose-dependent changes in phosphorylated Ser2 on RNAPII and a decrease in endogenous protein levels of MCL1 was evident in BCMP organoids after 6 h of treatment (Fig. 5h), indicating the drug was affecting transcription of endogenous genes as well as genetically introduced oncogenes. Among PDxO models, HCI-012 grew the fastest and was most sensitive to CDK9 inhibition (Fig. 5e, g), perhaps due to amplification of the *MYC* and *HER2* oncogenes.

## Discussion

Herein, we provide pre-clinical evidence demonstrating therapeutic efficacy of a recently developed, selective and orally bioavailable CDK9 inhibitor (CDDD11-8) in TNBC, using a panel of molecularly diverse human cell lines and patient-derived organoid models representing early and advanced disease. We show that treatment with CDDD11-8 dose-dependently reduced proliferation of cell line and organoid models at IC_50_ values within the nanomolar range, indicative of strong potency. We also provide the first RNAPII ChIP-seq data in a breast cancer context that demonstrates genome-wide promoter pausing following inhibition of CDK9. This genomic data accords with recent studies that demonstrate widespread RNAPII promoter pausing induced in cell line models of blood cancer upon treatment with different CDK9 inhibitors [45, 46]. Importantly, daily oral administration of CDDD11-8 was not associated with overt toxicity in mice at the doses tested. Our data at the 150 mg/kg/day dose accords with our recent pre-clinical study of acute myeloid leukaemia [30], but herein we additionally demonstrate lack of overt toxicity at a higher, 200 mg/kg/day, dose, including no effect on the proliferation of intestinal cells, which are particularly vulnerable to anti-cancer agents. We also found no effect on neutrophil numbers in the spleen, indicating the neutrophil reservoir was not depleted. This observation is important because neutropenia is a side effect of CDK9 inhibitors, including the only one (VIP152) to show clinical benefit to date [47, 48]. Using explants of non-malignant human breast tissues cultured ex vivo, we show that even at doses in the micromolar range, CDDD11-8 had no significant effect on tissue histology or the proliferative index of breast epithelial cells. While non-transformed breast epithelial cell organoids were inhibited by high doses of our CDK9 inhibitor, they were approximately 5-fold more resistant than their oncogene-transformed counterparts. These findings in normal mouse and human tissues were in striking contrast to the potent anti-proliferative, apoptotic effect of CDDD11-8 in the cancer cells and highlights the improved selectivity of this drug, a critical feature for targeting pan-essential genes like CDK9 in the treatment of cancer [49]. Indeed, implementation of CDK9 inhibition as a therapeutic strategy for cancer has been hampered by the poor selectivity and associated off target toxicity of CDK9 inhibitor drugs that have been clinically tested, predominantly in the context of blood cancer [15, 23, 29]. To date, no CDK9 inhibitors have been approved by the US Federal Drug Administration (FDA) [48], emphasizing the need for development and preclinical testing of new drugs like CDDD11-8.

Although CDDD11-8 dose-dependently reduced proliferation and triggered apoptosis in all TNBC cell lines, there were differences in potency among models. Intriguingly, the MFM-223 cell line was least and the MDA-MB-453 cell line most sensitive to CDK9 inhibition. These cell lines are both classified in the luminal-like subtype of TNBC [31]. The difference in their sensitivity to CDDD11-8 suggests that the molecular subtype of TNBC is unlikely to be a key determinant of response to CDK9 inhibition and that other factors may be more relevant. Intriguingly, while our least sensitive cell line had the highest basal level of MYC, our BCMP organoid model of ectopically driven *MYC* overexpression was highly sensitive to CDK9 inhibition, suggesting that a high level of MYC does not intrinsically confer treatment resistance. Rather, the BCMP organoid data and the *MYC* amplified PDxO models provide supporting evidence that *MYC* amplified breast cancers can be highly sensitive to this therapeutic approach. A multiplicity of tumour-intrinsic factors may influence relative response to CDK9 inhibition in TNBC [23]. For example, studies suggest that activity of a bromodomain protein (BRD4) or mediator complex protein (MED12) may dampen response to CDK9 inhibition [45, 50] and conversely that Protein Phosphatase 2 A (PP2A) activity [46] or the presence of wild-type P53 [51] may enhance response to this therapeutic strategy. Inhibition of the PIM3 kinase pathway has been shown to inhibit TNBC by indirectly targeting MYC [21] and to enhance response to a CDK9 inhibitor in models of lymphoma [45]. Hence, future work to develop biomarkers of response to CDK9 inhibition and to our CDDD11-8 drug specifically are warranted, as well as exploration of combinatorial strategies, with for example a PP2A agonist or PIM3 inhibitor, to enhance therapeutic efficacy.

Pre-clinical studies with Dinaciclib first implicated CDK9 as a therapeutic target for TNBC [27, 52], but this drug is not selective for CDK9 and had excessive off-target toxic side effects in clinical trials [24]. Recently, another new generation CDK9 inhibitor, Atuveciclib, was demonstrated to inhibit proliferation of the MDA-MB-453 and MDA-MB-231 cell line models of TNBC [26], a finding consistent with our results testing efficacy of CDDD11-8 in the same models. However, Atuveciclib was only efficacious at IC_50_ values in the micromolar range. Conversely, CDDD11-8 inhibited proliferation with IC_50_ values in the nanomolar range, indicating greater potency. Moreover, the effects of Atuveciclib have not been tested in vivo or using patient-derived models of TNBC as we have done herein. In recent years, other selective inhibitors of CDK9 have been described but to our knowledge have not been tested in the context of TNBC. In particular, the AZD4573 CDK9 inhibitor has demonstrated high selectivity and strong potency in haematological cancers but is not an orally bioavailable drug and treatment must be transient to prevent toxicity to normal cells [53, 54]. VIP152 has also shown promise pre-clinically and clinically in high-grade lymphomas and some solid tumours, administered weekly via injection [47, 48, 55]. The oral bioavailability of CDDD11-8 is a desirable feature, together with our data indicating low toxicity.

Our data supports CDDD11-8 as a selective inhibitor of CDK9, but we cannot definitively prove efficacy in TNBC is mediated by inhibition of this factor alone. While our previous characterization of CDDD11-8 indicated high selectivity (>50-fold) for CDK9 over other CDKs tested (−1, −2, −4, −6 and −7) [30], activity against other transcriptional CDKs (−8, −12, −13, −19) was not assessed. Studies have shown that CDK12 can also phosphorylate Ser2 on the RNAPII-CTD and thereby induce RNAPII promotor pausing [56], raising the possibility that CDDD11-8 may have activity via inhibition of CDK12. Selective inhibition of CDK12/13 has been shown to have efficacy in TNBC, but this predominantly occurred via inhibition of DNA repair genes [57]. In contrast, our data indicates that CDDD11-8 predominantly impacts MYC and E2F regulated genes, leading to significant downregulation of MYC and MCL-1 mRNA transcripts and corresponding proteins, which is more indicative of CDK9 inhibition. We also show that at nanomolar concentrations, CDDD11-8 reduced RNAPII phosphorylation at Ser2 in the CTD in all TNBC cell line models and, critically, induced genome-wide RNAPII promoter-proximal pausing. While RNAPII promoter-proximal pausing was widespread, CDDD11-8 treatment induced a cell cycle blockade at the G2/M phase, characteristic of preferential inhibition of CDK9 over other CDKs. This finding is consistent with a previous report that silencing CDK9, but not CDK1 or CDK2, led to a G2/M cell cycle arrest [27].

Our previous work also reported potent activity of CDDD11-8 against a limited number of other kinases, in particular the fms-like tyrosine kinase 3 (FLT3) protein, and its mutant form (FLT3-ITD), a known driver and therapeutic target in acute myeloid leukaemia [30]. FLT3 mutations are not common in any sub-type of breast cancer [6] and among sub-types, FLT3 expression is lowest in TNBC [58]. Hence, although our current study is unable to rule out a role for inhibition of FLT3, FLT3-ITD, or other classes of kinases in mediating therapeutic efficacy of CDDD11-8 in TNBC, this appears to be unlikely. Nevertheless, inhibition of these kinases may represent an interesting avenue to pursue in future studies.

To enhance clinical relevance of our findings, we investigated the effect of CDK9 inhibition with CDDD11-8 on five distinct patient-derived organoid models, which are more cellularly complex than cell lines and are being used for pre-clinical drug discovery [44]. Four of the organoid models used herein were developed directly from TNBC patient tissues, including two from chemo-resistant metastatic lesions, which alludes to the clinical relevance of our findings. Our in vivo work was also designed to increase clinical relevance by using the contemporary technique of mammary intraductal (MIND) xenografting to better mimic the breast tumour microenvironment where cancers normally develop [37, 38]. Treatment with CDDD11-8 significantly inhibited MIND tumour xenograft growth, was well tolerated, and did not cause histopathological changes in vital organs susceptible to off-target toxicities. While inhibition of MIND xenograft tumour growth was more modest than expected from the in vitro experiments, this likely reflects relative exposure to circulating drug because MIND tumours first develop within the mammary ducts and are not directly exposed to the vasculature until it invades the surrounding stroma. In contrast, tumours established in the mammary fat pad by conventional xenografting are more directly exposed to the vasculature and often become highly vascularised. A retrospective histological review indicated that MIND tumours in our experiments had not yet progressed to the invasive stage, being more representative of in situ rather than invasive lesions. Therefore, we hypothesize that a more potent therapeutic response would be achieved if treatment was initiated after progression to an invasive stage. Despite this limitation of the in vivo experiments, our data is encouraging and provides the necessary proof-of-principle for future in vivo and ex vivo studies incorporating a larger suite of preclinical (PDX, PDO, PDxO and PDE) models of TNBC in which our novel selective CDK9 inhibitor is administered alone or in combination with either standard-of-care chemotherapies or other emerging agents, such as a PP2A agonist [46], PIM3 inhibitors [21, 45], immunotherapies or PARP inhibitors [23], to enhance therapeutic efficacy. Such future studies would also facilitate development of a biomarker for patient selection to support clinical trials and ultimately ensure optimal therapeutic benefit.

In summary, this study supports CDK9 inhibition as a targeted therapeutic strategy for TNBC, for which none currently exists. Moreover, our findings warrant further development of CDDD11-8, a novel oral selective CDK9 inhibitor, for clinical evaluation in TNBC and potentially for other aggressive, highly proliferative cancers addicted to transcription.

## Methods

### Test compound

Development and initial characterization of our CDK9 inhibitor, CDDD11-8 (K_*i*_ = 8 nM), was described in [30]. For in vitro assays, a 10 mM stock solution was prepared in 100% dimethyl sulfoxide (DMSO) and stored at −20 °C. Drug was diluted in cell culture media on day of treatment. For in vivo experiments, CDDD11-8 was freshly formulated in 0.1 M sodium acetate buffer (pH 4.5).

### Cell lines

The MDA-MB-453 (HTB-131, RRID:CVCL_0418), MDA-MB-231 (HTB-26, RRID:CVCL_0062), MDA-MB-468 (HTB-132, RRID:CVCL_0419) and HEK 293 T/17 (CRL-11268G-1, RRID:CVCL_UE07) cell lines were obtained from the American Type Culture Collection (ATCC, USA) and the MFM-223 line (ACC-420, RRID:CVCL_1408) from the DSMZ-German Collection of Microorganisms and Cell Cultures (Germany, RRID:SCR_001711). All cell culture reagents were purchased from Sigma-Aldrich (USA). MDA-MB-453, MDA-MB-231, MDA-MB-468, and 293 T/17 cells were maintained in DMEM High Glucose medium supplemented with 10% (v/v) FBS and 2 mM L-Glutamine. MFM-223 cells were maintained in EMEM supplemented with 10% FBS, 2 mM L-Glutamine and 1x Insulin–Transferrin–Sodium Selenite Supplement. Cell lines were cultured at 37 °C in a humidified incubator containing 5% CO_2_, confirmed negative for mycoplasma contamination via testing with an IP-protected *Mycoplasma spp*. detection assay developed in house and authenticated by short tandem repeat profiling (CellBank Australia (RRID:SCR_013086)).

### Immunofluorescence

Cells were seeded at 70–80% confluence onto sterilized 22 mm^2^ coverslips in a 6-well plate then fixed in 10% neutral buffered formalin (10 min). Fixed cells were washed in PBS, permeabilized with 0.05% Triton X-100 (1 h), then incubated overnight at 4 °C with a CDK9 antibody (Cell Signalling Technology Cat# 2316, RRID:AB_2291505, 1:100) delivered in 10% goat serum/PBS blocking buffer. Cells were subsequently incubated in the dark at RT (1 h) with an Alexa Fluor® 488 Goat anti-Rabbit IgG (Life Technologies Cat# A-11029, RRID:AB_2534088, 1:400) secondary antibody, then with an Alexa Fluor® 568 Phalloidin (Invitrogen, Cat# A-12380, 1:400) primary antibody (20 min), followed by a DAPI (Thermo Fisher Scientific Cat# D1306, RRID: RRID:AB_2629482, 1:1000) nuclear counterstain (1 min). After washing, coverslips were mounted onto glass slides using fluorescent mounting media (DAKO) and sealed with nail varnish. Images were captured using an Olympus IX73 inverted optical fluorescence microscope (RRID:SCR_020346), using a 100X objective with immersion oil. Nuclei (DAPI), CDK9 (AF488) and Phalloidin (F-actin; AF568) were visualized using Ultra-violet (UV), Intermediate blue (IB) and Intermediate green (IG) long-pass filters, respectively.

### Plasmid construction and lentivirus production

The pDRM18, pDRM98, pDRM166, pDRM209, pDRM210, pJS137, and pJS299 plasmids were constructed by Gibson assembly using standard procedures. All use the MND promoter [59] to express multiple transgenes from a single open reading frame containing picornaviral 2 A linkages. pDRM18 (“LTN”) expresses the firefly luciferase gene (*luc*), E2A, tdTomato fluorescent protein, P2A, and the neomycin resistance gene (*aph*). pDRM98 (“mKate”) expresses nuclear localisation sequence (NLS)-tagged mKate2 fluorescent protein, P2A, and the blasticidin resistance gene (BSD). pDRM166 (“LKB”) expresses *luc*, P2A, the NLS-tagged mKate2 fluorescent protein, P2A and BSD. pDRM209 (“LTP”) expresses *luc*, E2A, tdTomato, T2A, and the puromycin resistance gene (*pac*). pDRM210 (“LGP”) expresses *luc*, E2A, enhanced green fluorescent protein (EGFP), F2A and *pac*. pJS137 (“HCM”) expresses the hygromycin resistance gene (*hph*), P2A, the *CCND1* open reading frame, T2A, and the *MYC* open reading frame with an N-terminal 3x hemagglutinin (HA) tag and a T58A activating mutation. pJS299 (“BBP”) expresses BSD, F2A, the *BMI1* open reading frame, E2A, and the *PIK3CA* open reading frame with an N-terminal 3x Flag tag and a H1047R activating mutation. Further details are available at Addgene (#174720–174723 & #183502). Lentiviral particles were produced by transfection of 293 T/17 cells with vector plasmid and packaging plasmids (psPAX2, Addgene, #12260, RRID:Addgene_12260; pMD2-G, Addgene, #12259, RRID:Addgene_12259) using polyethylenimine or calcium phosphate transfection as described [60]. Conditioned medium containing viral particles was harvested and concentrated ~200-fold using Vivaspin20 columns (GE Healthcare), according to manufacturer’s instructions. All breast cancer models used in this study were infected with concentrated lentivirus at a multiplicity of 1–2 infectious units per cell. Transduced cells were selected using the relevant antibiotic, supplemented into culture media at standard concentrations and durations as appropriate.

### Proliferation and apoptosis assays

Cell lines were transduced with lentivirus as described above to stably express nuclear-localized mKate2, enabling live cell imaging. Cells were plated at a density of 4–5 $\times$ 10^3^ cells per well in 96-well tissue culture plates (CoStar), with a minimum of 5 replicate wells per experimental condition. Following a 24 h incubation period, the media was replaced with media containing IncuCyte® Caspase-3/7 Green Apoptosis Assay Reagent (final concentration 5 µM, Sartorius, #4440). Concurrently, cells were treated with either vehicle (DMSO) or CDDD11-8. Plates were imaged on the Sartorius IncuCyte S3 Live Cell Analysis System (RRID:SCR_023147) for 5 d, capturing images in the red and green channels using a 10$\times$ objective. Resultant images were analysed to determine the number of live (red; mKate2) and dead (green; Caspase-3/7) cells using the associated IncuCyte S3 software. Image analysis was trained using six representative images from both low and high confluence, vehicle and CDDD11-8 treated cells. Three independent proliferation assays were conducted for each cell line to determine a robust half-maximal inhibitory concentration (IC_50_).

### Cell cycle analyses

Cell lines were treated with vehicle or CDDD11-8. Following 3–5 d of treatment, cells were washed once and fixed in 70% ethanol at 4 °C overnight. Fixed cells were washed once with DPBS and stained for 30 min at RT with propidium iodide (PI) solution (50 μg/mL PI, 100 µg/mL RNase A, 0.1% Triton X-100). At least 1 × 10^5^ PI-stained cells were sorted using a FACS Canto II with BD CellQuest Pro software (BD Biosciences, RRID:SCR_014489) using standard procedures. Data was processed using FlowJo v10.6 (RRID:SCR_008520). Each graph was scaled to the mode, corresponding to cells in G1 phase. A minimum of two independent experiments were performed for each cell line, with three technical replicates per condition.

### Western blotting

Cell lines were seeded in 6-well tissue culture plates (Corning) at ~70% confluency and allowed to attach for 48 h before treatment with vehicle or CDDD11-8 for 6 h. Cells were harvested in RIPA buffer (50 mM Tris-HCl pH 8.0, 150 mM NaCl, 0.5% sodium deoxycholate, 0.1% SDS, 0.1% Triton X-100) supplemented with 1x cOmplete Protease Inhibitor (Roche) and 1x HALT Phosphatase Inhibitor (Thermo Scientific). Protein concentration was quantified with a Pierce BCA protein assay (Thermo Scientific). Protein lysates (40 µg) were denatured in 1x loading dye (0.27 M Tris, 10.3% SDS, 6% β-mercaptoethanol, 35% glycerol, and 0.05% Bromophenol blue) at 95 °C for 5 min then loaded into 4–12% Bis-Tris gradient SDS-PAGE gels run with 1x MOPS buffer (BIO-RAD). Immunoblotting was performed using standard protocols and nitrocellulose membranes (GE) probed with the following primary antibodies: CDK9 antibody (Cell Signalling Technology Cat# 2316, RRID:AB_2291505, 1:1,000), RNA Pol II CTD repeat YSPTSPS (Abcam Cat# ab817, RRID:AB_306327, 1:1,000), RNA Pol II CTD repeat YSPTSPS phospho Ser2 (p-Pol II Ser2, Abcam Cat# ab193468, RRID:AB_2905557, 1:5000), c-MYC (Cell Signalling Technology Cat# 9402, RRID:AB_2151827, 1:1,000), MCL-1 (Cell Signalling Technology Cat# 5453, RRID:AB_10694494, 1:1000) and GAPDH (Millipore Cat# MAB374, RRID:AB_2107445, 1:2000). Detection of primary antibodies was performed using HRP-conjugated anti-mouse (DAKO Cat# P0161, RRID:AB_2687969, 1:1,000) or anti-rabbit (DAKO Cat# P0448, RRID:AB_2617138, 1:1,000) secondary antibodies as appropriate. Signals were visualized with Clarity Western ECL Substrate using a ChemiDoc MP imaging system (BIO-RAD, RRID:SCR_019037). Densitometry was performed on unsaturated images using ImageLab software (BIO-RAD) and normalized to GAPDH. Experiments were performed in triplicate and two independent experiments were performed for each cell line.

### RNA isolation and quantitative RT-PCR

Cell lines were treated with vehicle or CDDD11-8 for 4 h prior to harvest and RNA isolated with TriReagent (Sigma-Aldrich) using manufacturer protocols. RNA was DNase treated using the Turbo DNA-Free Kit (Thermo Fisher Scientific) and quantified using a Nanodrop 1000 spectrophotometer (Thermo Fisher Scientific). Reverse transcription was performed on 1 µg RNA using the iScript Select cDNA Synthesis Kit (BIO-RAD). Quantitative real time RT-PCR was conducted with iQ SYBR Green Supermix (BIO-RAD) using the CFX384 Real-Time PCR Detection System (BIO-RAD). Gene expression was determined by the 2^-ΔΔCt^ method and normalized to *GAPDH* expression. Each condition was represented in triplicate and two independent experiments were performed for each cell line.

### Chromatin immunoprecipitation and associated bioinformatics analyses

Chromatin immunoprecipitation (ChIP)-seq experiments were performed as previously described [61]. In brief, MDA-MB-453 cells were seeded at ~75% confluency into 15 mm^2^ culture dishes and incubated for 3 days before treatment (4 h) with vehicle or CDDD11-8 (600 nM). Two biological replicate experiments were performed representing consecutive passages of cells. Each ChIP was performed with 10 µg RNA Polymerase II antibody (Santa Cruz Biotechnology Cat# sc-47701, RRID:AB_677353) and 100 µL Protein A Dynabeads (Invitrogen). Recovered DNA was amplified using the NEBNext Ultra II DNA Library Prep Kit (NEB) following manufacturer’s protocols. DNA libraries were sequenced on the Illumina NovaSeq 6000 Sequencing System (RRID:SCR_016387) to a minimum of 60 million 150 bp paired-end reads per sample. Reads were aligned to human assembly GRCh37 (hg19) using Bowtie 2 (RRID:SCR_016368) [62]. RNAPII peaks were called for each sample with MACS2 (RRID:SCR_013291) [63] using default settings, against a paired chromatin input. Peaks were annotated to UCSC.hg19.knownGene, and consensus peaks (representing peaks called in both replicates per condition) intersecting promoters (±300 bp of a transcription start site) were subject to differential enrichment analysis using DiffBind (RRID:SCR_012918) [64]. Differentially enriched promoters were defined using an FDR of < 0.05. In the case of multiple transcripts, peaks associated with the longest isoform were retained. Replicate Spearman correlation plots, read density plots, and heatmaps were generated with deepTools (RRID:SCR_016366) [65], using the public server at usegalaxy.org (RRID:SCR_006281) [66]. Genome coverage was visualized using Integrative Genomics Viewer (IGV, RRID:SCR_011793) [67]. Pausing index analyses were performed as described [68]. For each transcript, enrichment at the proximal promoter (defined over a window 50 bp upstream and 300 bp downstream of the TSS) was compared to enrichment over the entire gene body (defined as 300 bp downstream of the TSS to 3 kb past the TES). Genes <3 kb from each other, or those <1 kb in length were excluded from analysis. The pausing index was calculated as the log_2_ ratio between the read count per length of the proximal promoter over the read count per length of the gene body. A two-sided, paired Wilcoxon test was used to compare the RNAPII pausing index between Vehicle and CDDD11-8 treatment. Gene ontologies were performed with Goseq (RRID:SCR_017052) [69] and the HALLMARK gene set from the Molecular Signatures Database (RRID:SCR_016863) [70]. A hypergeometric distribution was used to analyse RNAPII promoter peaks gained with CDDD11-8 treatment against a background of all unchanged or decreased promoter peaks. Significance was determined using an FDR < 0.05. Homer de novo motif analysis (RRID:SCR_010881) was performed on differential peaks (CDDD11-8 vs DMSO).

### Patient-derived breast organoid models

All experiments involving patient-derived organoids (PDOs) of breast tissue were conducted in accordance with national and international ethical guidelines on human and animal research. Tissue was collected from women undergoing breast surgery after providing informed, written consent (Approval #AC-2017–3070, MESR, France). The BCMP model is a genetically defined organoid model derived from normal human breast tissue obtained from a reduction mammoplasty surgery and engineered to overexpress four oncogenes (*BMI1*, *CCND1*, *MYC*^T58A^, and *PIK3CA*^H1047R^). Organoids of genetically unmodified breast epithelial cells were also generated from two independent reduction mammoplasty tissue samples. The MgA1 model was derived from a patient with a small TNBC tumour surrounded by pre-malignant microglandular adenosis. Tissue digestion and lentiviral transduction were performed as described [71]. Briefly, fresh surgical specimens were transferred to the laboratory in tissue culture medium, then macroscopically dissected and minced into <1 mm^3^ pieces. Tissue fragments were digested at 37 °C with 1 mg/mL collagenase type IV (Gibco) in organoid medium containing 10 µM Y-27632 Rho kinase inhibitor (Selleck) in a 6-well tissue culture plate, with occasional mixing by pipetting. Digestion was monitored using a phase contrast microscope (Olympus) and deemed complete when the tissue was reduced to small clusters of cells free of collagen fibrils. Cell-free DNA was then digested for <5 min with 100 µg/mL DNase I (Sigma), after which cell clusters were washed three times in Advanced DMEM/F-12 (Gibco) containing 10 µM Y-27632. For BCMP, the dissociated cell population was then enriched for mammary epithelial cells using the EasySep Human EpCAM Positive Selection Kit II (Stem Cell Technologies), following manufacturer’s protocols.

Lentiviral infections were performed in Ultra-Low Attachment plates (Corning) by overnight incubation at 37 °C and 5% CO_2_. After infection, cells were washed several times to remove residual lentivirus. BCMP was infected with the LGP (pDRM210) lentiviral vector for monitoring in vivo growth by *luc* bioluminescence, in vitro growth by GFP fluorescence, and to allow for selection of transduced cells with puromycin. BCMP was also infected with the HCM (pJS137) and BBP (pJS299) lentiviral vectors to allow stable overexpression of BMI1, CCND1, MYC^T58A^, and PIK3CA^H1047R^ oncogenes, and to allow for selection of transduced cells with hygromycin and blasticidin. MgA1 was infected with the LTN (pDRM18) lentiviral vector for monitoring in vivo growth by measuring bioluminescence (*luc*), and monitoring of in vitro growth by measuring tdTomato fluorescence, and to allow for selection of transduced cells with hygromycin and neomycin. The MgA1 model was initially propagated inside the mammary ducts as a patient-derived xenograft (PDX) in NSG mice (Approval #4033, MESR, France) using methodology described below. A palpable tumour formed after 6 months in the first xenograft passage, and after 3 months in the second passage. The MgA1 organoid line was derived from the second in vivo xenograft passage by repeating the digestion process described above on the excised tumour.

Organoids of advanced breast cancer generated from established patient-derived xenografts (PDXs), designated PDxO, were obtained from the Huntsman Cancer Institute (HCI) Preclinical Research Resource core facility. Clinically, the HCI-010 model represents a pre-treated metastatic basal-like TNBC lesion, the HCI-012 model a pre-treated metastatic HER2-amplified lesion and the HCI-016 model a metastatic basal-like TNBC lesion of unknown treatment history as previously described [44]. PDxO models were infected with the LKB (pDRM166) lentiviral vector for monitoring in vivo growth by *luc* bioluminescence, in vitro growth by mKate2 fluorescence, and to allow for selection of transduced cells with blasticidin.

### Patient-derived breast organoid culture

PDOs and PDxOs were suspended in 100% Matrigel (Corning) and plated in tissue culture plates (Corning) in domes of up to 20 µL each. Domes were allowed to solidify by plate inversion for 30 min at 37 °C then overlaid with organoid medium and cultured at 37 °C and 5% CO_2_. The BCMP and normal breast epithelial cell organoid medium contains Advanced DMEM/F-12 (Gibco) supplemented with 0.1 mg/mL Primocin (Invivogen), 1x GlutaMax (Life Technologies), 10 mM HEPES (Life Technologies), 10% R-Spondin-1 conditioned medium (in house), 10% Noggin-conditioned medium (in house), 1.25 mM N-Acetyl-L-cysteine (Sigma), 10 mM Nicotinamide (Sigma), 0.5 μM A83-01 (R&D), 1x B27 (Life Technologies), 1 µM PGE2 (R&D), 0.5 μM SB202190 (Sigma), 5 nM Heregulinβ−1 (PeproTech), 5 ng/mL FGF7 (PeproTech), 20 ng/mL FGF10 (PeproTech), 10 ng/mL Amphiregulin (PeproTech), and 10 µM Y-27632 (Selleck). Medium for MgA1, HCI-010 and HCI-016 organoids contains Advanced DMEM/F-12 (Gibco) supplemented with 0.1 mg/mL Primocin (Invivogen), 1x GlutaMax (Life Technologies), 10 mM HEPES (Life Technologies), 5% FBS (Sigma), 10 ng/mL human EGF (Sigma), 1 µg/mL hydrocortisone (Sigma), and 10 µM Y-27632 (Selleck). HCI-012 organoid culture medium is identical that used for HCI-010 and HCI-016, with the addition of 10 nM Heregulin β1 (PeproTech). Organoid medium was replaced 2–3 x weekly, and PDOs or PDxOs were passaged when their growth began to plateau, as indicated by fluorescence intensity. BCMP, HCI-010, HCI-016 and HCI-012 models were passaged by culture media aspiration, followed by Matrigel digestion using TrypLE (Life Technologies) for 3–4 min. MgA1 was passaged by spiking 0.25 U/mL Dispase I (Sigma) into the culture media, pipetting the Matrigel vigorously, and incubating at 37 °C until organoids had been completely digested away from the Matrigel (~3 h). After TrypLE or Dispase treatment, organoids were centrifuged and washed in Advanced DMEM/F-12 + 10 µM Y-27632, ahead of resuspension and plating in an appropriate volume of cold Matrigel as described above. MgA1 was split using a ratio of 1:1–2, BCMP was split at a ratio of ~1:8, and PDxO models were split at a ratio of 1:5–6. PDO/PDxO growth rate was routinely monitored by manual fluorescent imaging with a 2X objective on an Olympus IX71 microscope, with the total fluorescent intensity quantified using Fiji (ImageJ). The BCMP and cancer-derived organoids were established and used within 15 serial passages whereas organoids of normal breast epithelial cells were established and used within 1–3 passages.

### Organoid proliferation assays

Organoids were plated in 2 µL Matrigel domes in 96-well tissue culture plates (Corning), with a minimum of 8 replicate wells per condition. After plating, organoids were cultured for a minimum of 24 h then treated with vehicle or CDDD11-8. Treatments were renewed every three days. BCMP and PDxO organoid growth was monitored using live cell imaging (Incucyte S3, Sartorius) at 4X magnification, capturing red (MgA1, HCI-010, HCI-012, HCI-016) or green (BCMP) fluorescence. The total fluorescent intensity was calculated as a fold change in fluorescent intensity relative to time 0 for each Matrigel dome. Since organoids of normal breast epithelial cells were not labelled with mKate, viability was assessed using a CellTiter-Glo 3D Cell Viability Assay (Promega, # G9681) on Day 0 and Day 10 of treatment and luminescence quantified using a Fluostar Omega microplate reader (BMG Labtech). A minimum of two independent experiments were performed for each organoid model to ensure data represents a consistent response.

### Ex vivo culture of patient-derived explants of normal human breast tissues

Normal, non-malignant human breast tissues were collected following informed consent from women undergoing breast reduction surgery at the Flinders Medical Centre, Adelaide, South Australia (#H-2015-175). Tissues were cultured ex vivo as patient-derived explants (PDEs) as previously described [40, 72]. In brief, glandular tissue was macroscopically dissected into ~1 mm^3^ pieces and randomly placed onto gelatine dental sponges (Ethicon) pre-soaked in culture media into a 24-well tissue culture plate (Corning). Wells were then filled with 500 µL culture media containing phenol red-free RPMI-1640 media (Gibco) supplemented with 10% FBS, 10 µg/mL human recombinant insulin (Sigma), 10 µg/mL hydrocortisone (Sigma), and 1X anti-mycotic/anti-biotic (Sigma). PDEs were incubated on sponges at 37 °C with 5% CO_2_. After 24 h pre-culture, the medium was replaced with fresh culture medium supplemented with vehicle or CDDD11-8, followed by culture for a further 48 h. This time frame was sufficient to induce changes to the viability and proliferative capacity of breast epithelial cells within PDEs based on pilot studies of a non-selective CDK9 inhibitor (CDKI-73) with similar structure to CDDD11-8 (data not shown), consistent with an independent study showing uptake of a small molecule non-steroidal inhibitor into similarly cultured prostate PDEs after 6 h, with maximal effect at 48 h of treatment [73]. Four tissue pieces from each reduction mammoplasty case were randomly allocated to each treatment on a single sponge. Cultured PDEs were fixed in 10% neutral-buffered formalin at 4 °C overnight, and subsequently paraffin-embedded as per standard protocols.

### Mammary intraductal (MIND) xenografts

Animal experiments were approved by the University of Adelaide Animal Ethics Committee (#M-2018-088). Female NOD.Cg-Prkdc^scid^ Il2rg^tm1Wjl^/SzJ (NSG) mice, aged 8–12 weeks old, were socially housed in individually ventilated cages, in temperature- and light cycle-controlled rooms located within the specific pathogen-free Adelaide Health and Medical Sciences Biomedical Research Facility. Mice were provided with *ad libitum* access to food, water, and nesting materials, and were monitored for general wellbeing at least once daily, according to a clinical record sheet.

The MDA-MB-453 cell line was infected with LTP (lentiviral vector pDRM209) and selected using puromicin, while the MDA-MB-468 cell line was infected with LKB (lentiviral vector pDRM166) and selected using blasticidin, to enable bioluminescent monitoring of in vivo xenograft growth. Transduced cell lines were confirmed as negative for residual lentivirus by p24 ELISA (Takara Bio) prior to in vivo use. MDA-MB-453 (2 × 10^5^) or MDA-MB-468 cells (2 ×10^5^) were resuspended in 10 µL media and injected into the fourth inguinal mammary ducts of NSG mice (*n* = 20) as described [37, 38]. For the MDA-MB-453 experiment, mice were injected unilaterally 5 d post-injection and allocated by simple randomization to receive either vehicle (0.1 M sodium acetate, pH 4.5; n = 10 mice) or CDDD11-8 (150 mg/kg/day; *n* = 10), delivered daily by oral gavage for 15 consecutive days. For the MDA-MD-468 experiment, mice were injected bilaterally and randomized for treatment 19 days post-injection, receiving vehicle (*n* = 5) or CDDD11-8 (200 mg/kg/day; *n* = 5) delivered daily by oral gavage for 15 consecutive days. Tumour growth was monitored using the IVIS Lumina X5 In Vivo Imaging System (Perkin Elmer, RRID:SCR_020397). IVIS imaging was conducted five days after injection to confirm the presence of tumour cells and every six days after treatment commencement. Bioluminescence (photons/sec) was quantified using a standardized region of interest size for each image. Following 15 d treatment, organs (spleen, liver, and xenografted mammary glands) were harvested, formalin fixed, and paraffin embedded (FFPE) as per standard protocols.

### Histology and immunohistochemical staining

FFPE tissue blocks were sectioned on the RM2235 manual rotary microtome (Leica) at 4 µm. Sections were baked onto adhesive microscope slides (TRAJAN) for a minimum of 60 min at 60 °C before xylene de-paraffinisation and dehydration with 100% ethanol. Histology slides were stained with Lillie-Mayer’s haematoxylin (Australian Biostain), differentiated with 0.3% acid alcohol solution, and briefly counterstained in 1% alcoholic eosin/phloxine (Australian Biostain). For immunohistochemistry, slides were incubated in 0.9% hydrogen peroxide (Chem-Supply) to quench endogenous peroxidase activity and then subjected to heat‐induced epitope retrieval in 10 mM citrate buffer (pH 6.5) within a Decloaking Chamber (BioCare Medical). Slides were subsequently blocked for 10 min with Avidin/Biotin kit (Invitrogen) and then with 5% goat serum for 30 min, followed by incubation with a Ki-67 antibody (Agilent Cat# M7240, RRID:AB_2142367, 1:400) or a myeloperoxidase (MPO) antibody (Agilent Cat# A039829-2, RRID:AB_2335676, 1:1000) overnight at 4 °C inside a humidified chamber. Slides were then incubated with biotinylated secondary antibody (Goat Anti-Mouse Immunoglobulins/Biotin, Agilent Cat# E0433, RRID:AB_2687905, 1:400 for Ki-67; Goat Anti-Rabbit Immunoglobulins/Biotin, Thermo Fisher Scientific Cat# 31823, RRID:AB_228345, 1:500 for MPO), and subsequently with streptavidin-conjugated tertiary antibody (Streptavidin/HRP, Agilent, #P0397, 1:500), each for 1 h at RT. Stained slides were developed using 3‐3′‐diaminobenzidine chromogen (Sigma), and counter-stained with hematoxylin prior to mounting. Appropriate positive and negative controls were included in all assays. Slides were scanned using a NanoZoomer Digital Slide Scanner (Hamamatsu, RRID:SCR_022537). Ki67 staining was quantified on PDEs by manual counting of all fields containing epithelial cells (*n* = 500 – 3,000 counted cells per specimen). MPO staining was quantified using QuPath software (RRID:SCR_018257), in which positive cells were scored in entire tissue sections representing the spleens of mice from Vehicle (*n* = 5) and CDDD11-8 (*n* = 5) treated mice.

### Statistical analyses

Cell line and organoid proliferation assays (and apoptosis assays, where relevant) were analysed using a two-way repeated measures ANOVA, followed by Dunnett’s multiple comparison test. IC_50_ values were determined for each independent proliferation assay using a four-parameter logistic function. IC_50_ values for each cell line, representing three independent experiments, were compared using an ordinary one-way ANOVA followed by Tukey’s multiple comparisons test. Cell cycle and RT-PCR data was analysed using an ordinary two-way ANOVA followed by Dunnett’s multiple comparisons test, comparing each CDDD11-8 dose to that of the vehicle. Organoid IC_50_ data was compared between models using a sum-of-squares F-test. Tumour xenograft growth data was analysed after log transformation using a two-way repeated measures ANOVA followed by Šídák’s multiple comparisons test, with nonlinear least squares regression model (curve fit) to determine the line of best fit for each treatment group. Endpoint (day 15) mouse bodyweight data was matched to treatment entry weight (day 0) for each mouse and compared between treatment groups using an unpaired, two-sided t-test with Welch’s correction. PDE data was analysed using a one-way repeated measures ANOVA followed by Dunnett’s multiple comparisons test. Unless specified, all data were analysed with Geisser-Greenhouse correction (no assumption of equal variance). All data are expressed as the mean ± S.E.M. A value of *p* < 0.05 was considered statistically significant unless otherwise stated.

### Supplementary information

Supplementary Figure Legends

Supplementary Figure 1

Supplementary Figure 2

Supplementary Figure 3

Supplementary Figure 4

Supplementary Figure 5

Supplementary Table 1

## Data Availability

All ChIP-seq data are deposited in the Gene Expression Omnibus (RRID:SCR_005012) under accession number GSE184335.
