# Supplementary material for: Selective inhibition of CDK9 in triple negative breast cancer
Source: Oncogene. 2023 Nov 24;43(3):202–15. doi: 10.1038/s41388-023-02892-3 (PMC10786725; doi:10.1038/s41388-023-02892-3)
Supplement: Supplementary file 1 — Supplementary Figure Legends [file 41388_2023_2892_MOESM1_ESM.docx]

**Supplementary Figure Legends**

**Supplementary Figure 1: CDDD11-8 inhibits TNBC cell line proliferation, inducing G2/M arrest. (a)** Bar plot showing the endpoint half-maximal inhibitory concentration (IC_50_) for CDDD11-8 across four TNBC cell line models. Data reflects the mean ± S.E.M. of three independent experiments per cell line, each containing five technical replicates per condition, performed as described in Figure 1. IC_50_ values were determined for each independent experiment using a four-parameter logistic function and compared using an ordinary one-way ANOVA (*F* = 42.55, d.f. = 3, *p* < 0.0001). Significant differences (defined as *p* < 0.05) between cell line IC_50_ values are determined using Tukey’s multiple comparisons test. The average IC_50_ value is inset into each graph. **(b)** Representative propidium iodide-based FACS histograms depicting the change in cell cycle stage in TNBC cell lines after treatment with CDDD11-8 (300 nM; MDA-MB-453 cells, 600 nM; MDA-MB-468, MDA-MB-231, and MFM-223 cells). Vehicle and CDDD11-8 histograms are overlaid and scaled to the mode. Cell cycle phases are annotated within the left-most histogram, with G2/M phase highlighted in grey. MDA-MB-453, MDA-MB-468 & MFM-223 were treated for 3 d, while MDA-MB-231 cells were treated for 5 d. Asterisks denote a significant difference between treatment and vehicle, as determined using Dunnett’s multiple comparisons test. **p* < 0.05; ***p* < 0.01; ****p* < 0.001; *****p* < 0.0001. **(c)** DepMap data for CDK9 gene deletion (CRISPR) and suppression (RNAi) in TNBC cell lines. ^1^CRISPR and RNAi gene scores are ranked by Z-score with 0.0 being no effect on cell viability. A score of -0.5 or higher indicates depletion whereas a score of -1.0 or higher indicates dependency (i.e., kills cells).

**Supplementary Figure 2: CDDD11-8-mediated changes in RNA Polymerase II chromatin enrichment.** **(a)** Dot plots demonstrating the genome-wide correlation between two replicate RNA Polymerase II (Pol II) ChIP-seq experiments conducted on independent passages of MDA-MB-453 cells after 4 h treatment with vehicle or CDDD11-8 (600 nM). Data is associated with Figure 3. Spearman correlation coefficients are inset within each graph and demonstrate high correlation (*R* > 0.93) between replicates within a given condition. **(b)** Input-normalized read density plots (top panels) and heatmaps (bottom panels) for data in **(a)** showing a global increase in Pol II ChIP-seq enrichment within promoter regions across the genome. Pol II data is presented as the average log-transformed enrichment over the input control, and corresponds to data from two replicates, representing independent passages of cells. TSS; transcription start site, TES; transcription end site. **(c)** Volcano plot for differentially enriched Pol II promoters described in Figure 3b, showing a significant increase in Pol II enrichment at promoters after treatment with CDDD11-8. Each data point represents an individual promoter and coloured according to differential enrichment status (blue; FDR > 0.05, red; FDR < 0.05). Selected promoters are annotated to their corresponding gene. **(d)** Word clouds associated with Figure 3d, depicting promoters across the top three over-represented HALLMARK gene sets which increase in Pol II enrichment after CDDD11-8 treatment. Parentheses indicate the number of differentially enriched promoters found within each gene set. The font size for each promoter is arranged in order of decreasing FDR.

**Supplementary Figure 3: Mammary intraductal xenograft studies. (a)** Representative images showing negative controls for MCL-1 immunohistochemistry in MDA-MB-453 xenograft tumours presented in Figure 4. **(b)** Left panel: Tumour growth curves assessed using *in vivo* bioluminescence of MDA-MB-468 cells expressing luciferase. Data was analysed using a two-way repeated measures mixed-effects ANOVA (Interaction: *F* = 3.052, d.f. = 4, *p* = 0.0228) and least squares regression (Vehicle: R^2^ = 0. 5252, *k* = 0. 05490; CDDD11-8: R^2^ = 0. 3495, *k* = 0.04971). Right panel; Representative bioluminescent images of mice growing bilateral MDA-MB-468 MIND xenograft tumours at endpoint after treatment with vehicle or CDDD11-8. Asterisk indicates a statistically significant difference, **p* < 0.05. **(c)** Bodyweight changes of NSG mice (n = 5 per group, injected bilaterally) over 16 d during daily oral gavage treatment with either a vehicle or CDDD11-8 (200 mg/kg/day). Data was analysed using a two-way repeated measures ANOVA (*p* = 0.4645). **(d)** Representative Ki67 staining images of mouse jejunum after 16 days daily treatment with either a vehicle or CDDD11-8. Scale bars = 50 µm.

**Supplementary Figure 4: CDDD11-8 does not inhibit proliferation of *ex vivo* cultured patient-derived normal breast tissues. (a)** Graphical representation of the *ex vivo* culture of normal patient-derived breast tissue explants. **(b)** Quantification of Ki-67 positivity in normal breast PDEs (n = 4 independent cases) showing that increasing doses of CDDD11-8 do not change Ki-67 positivity after 48 h treatment. For each case, matched Ki67 positivity for each dose of CDDD11-8 was analyzed using a one-way repeated measures ANOVA (*F* = 0.1885, d.f. = 3, *p* = 0.8386). **(c)** Representative H&E staining (upper panel) and Ki-67 immunohistochemistry (lower panel) in normal breast tissue PDEs after 48 h treatment with either vehicle or 2.7 µM CDDD11-8. Scale bars = 50 μm. **(d)** Reduction mammoplasty breast epithelial cell organoid viability as measured by CellTitre Glo 3D after 10 days treatment with increasing doses of CDDD11-8. Asterisks denote a significant difference between treatment and vehicle, as determined using Dunnett’s multiple comparisons test. ***p* < 0.01; *****p* < 0.0001.

**Supplementary Figure 5: Histology of MgA1, a TNBC arising from a region of microglandular adenosis. (a)** Immunohistochemical staining results in the patient and after establishment of a patient-derived xenograft (PDX). The sample in the patient was surrounded by microglandular adenosis. Scale bars = 100 µm.
