# Supplementary figures and images for: Selective inhibition of CDK9 in triple negative breast cancer
Source: Oncogene. 2023 Nov 24;43(3):202–15. doi: 10.1038/s41388-023-02892-3 (PMC10786725; doi:10.1038/s41388-023-02892-3)

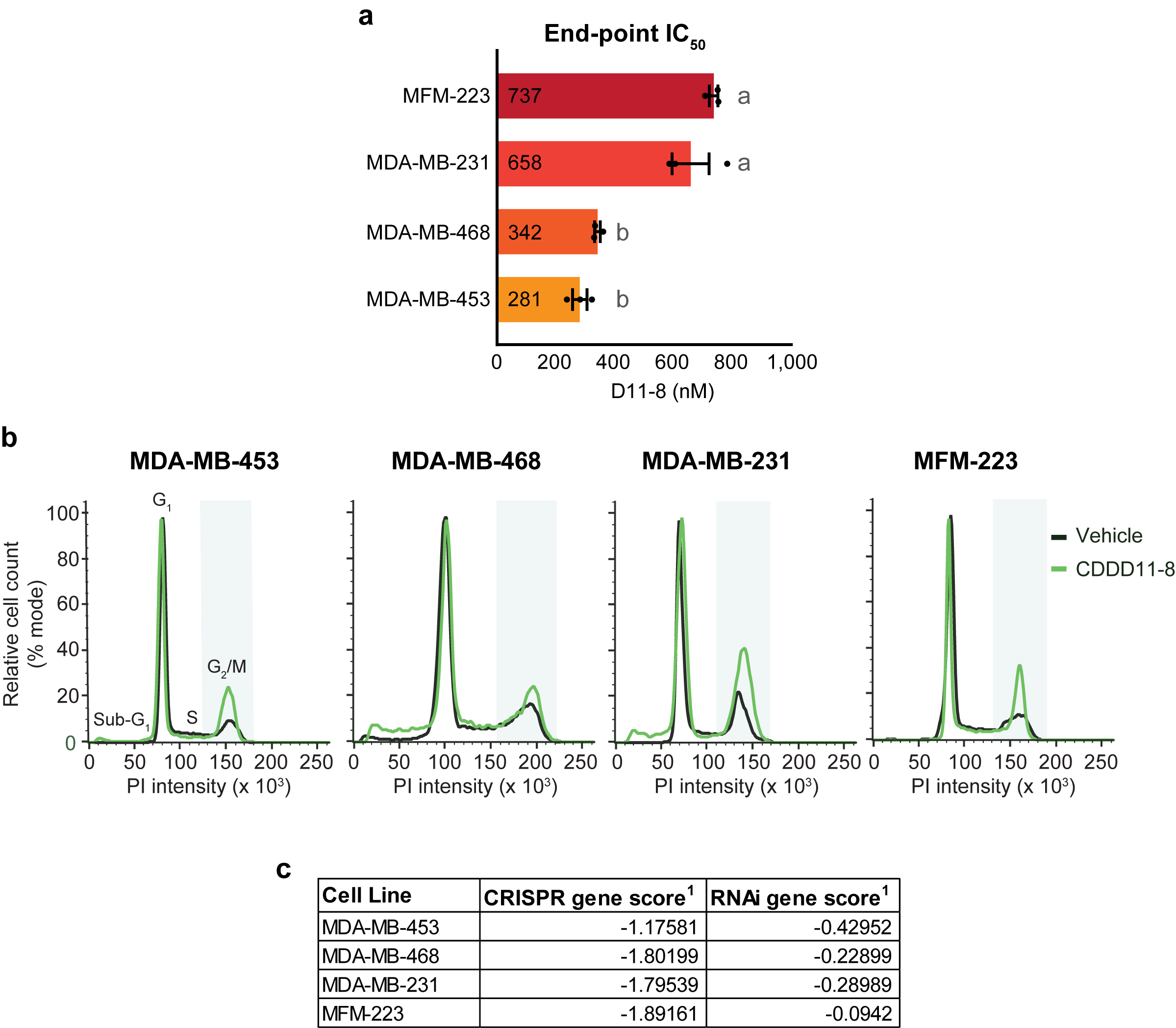

Supplement: Supplementary file 2 — Supplementary Figure 1 [file 41388_2023_2892_MOESM2_ESM.tif]

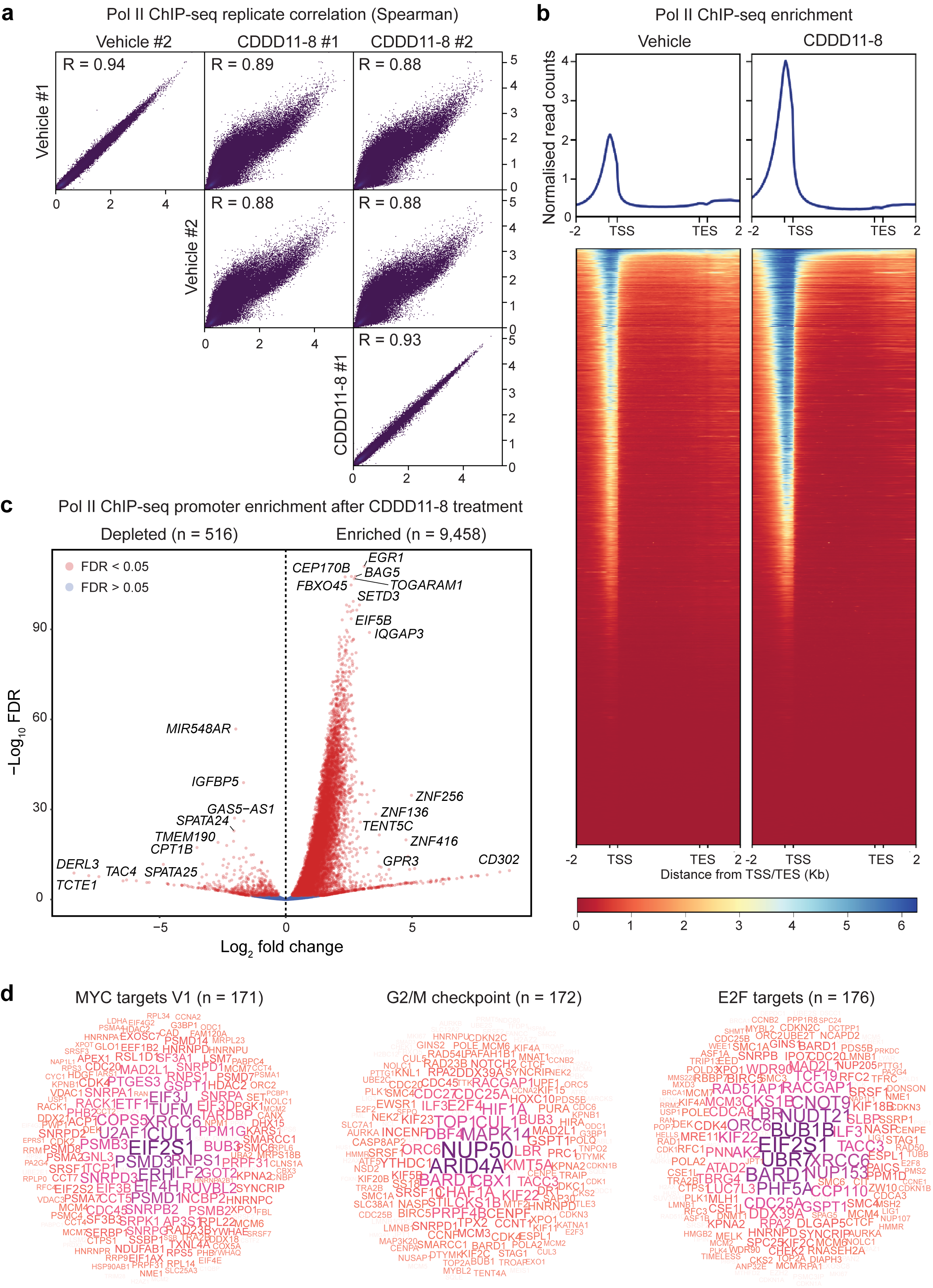

Supplement: Supplementary file 3 — Supplementary Figure 2 [file 41388_2023_2892_MOESM3_ESM.tif]

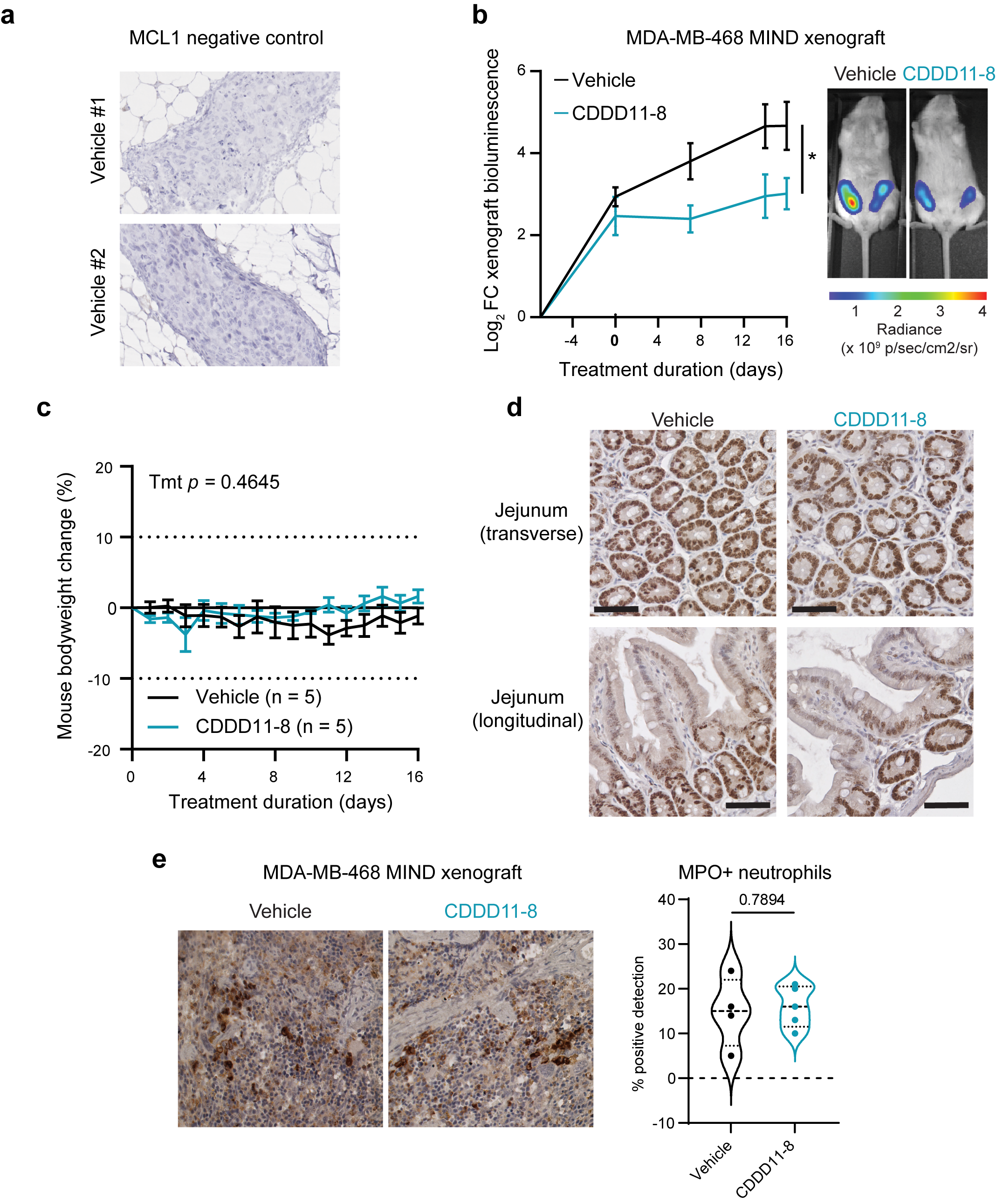

Supplement: Supplementary file 4 — Supplementary Figure 3 [file 41388_2023_2892_MOESM4_ESM.tif]

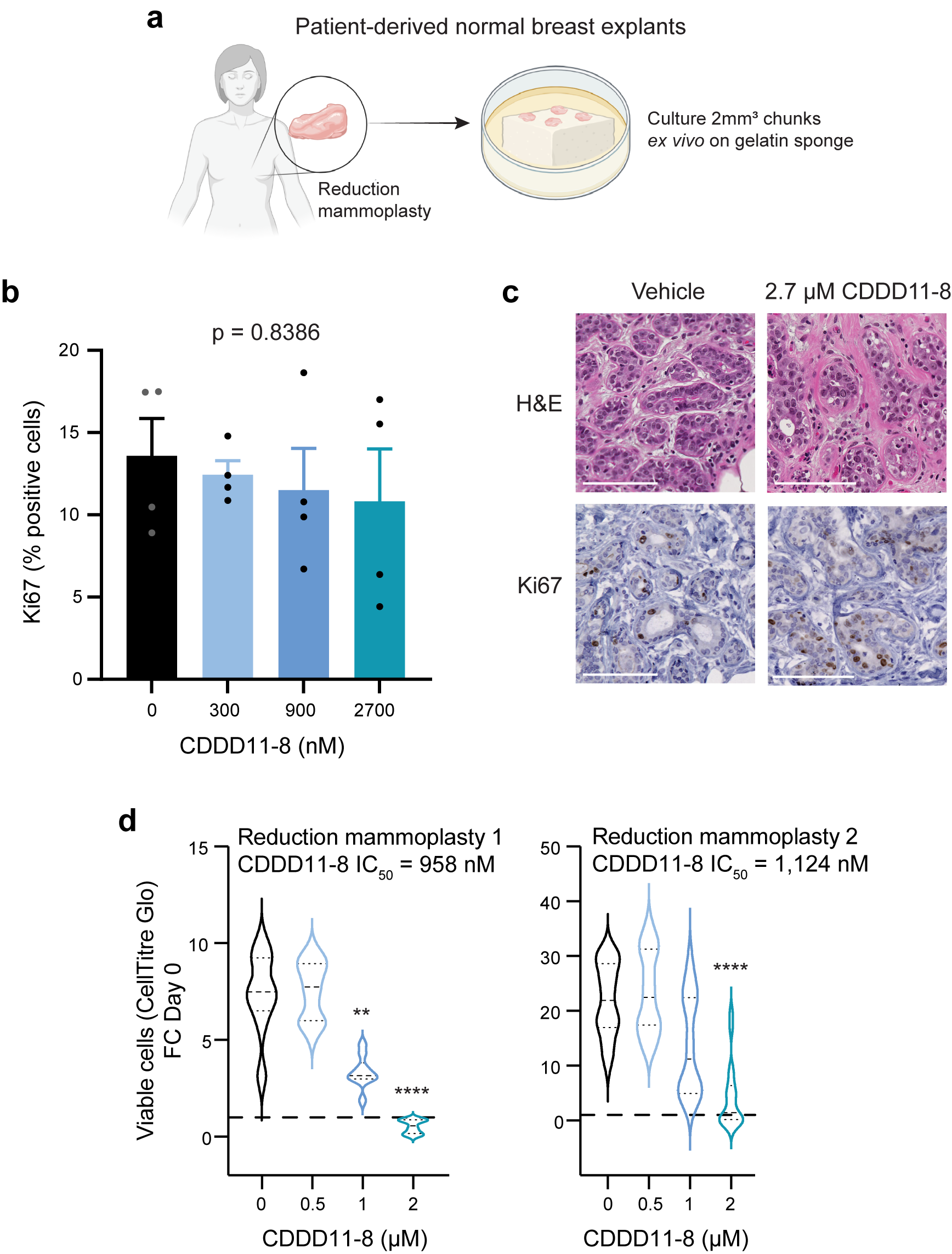

Supplement: Supplementary file 5 — Supplementary Figure 4 [file 41388_2023_2892_MOESM5_ESM.tif]

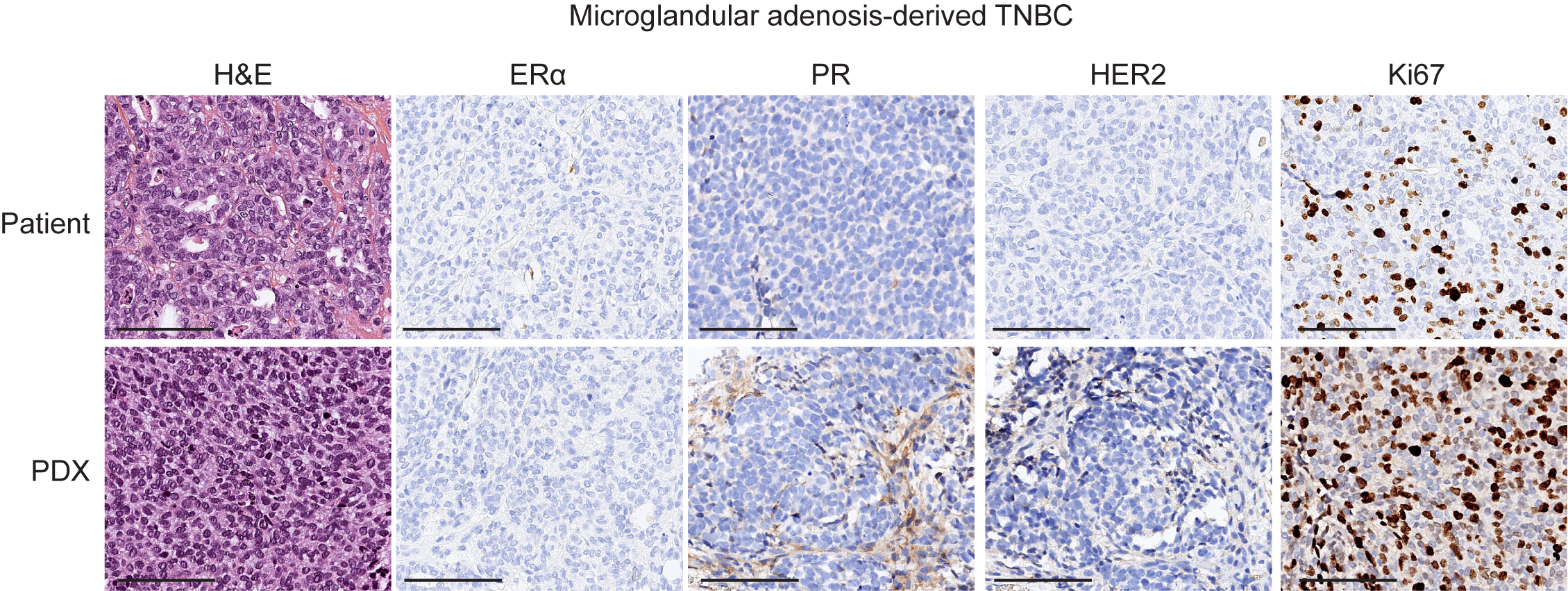

Supplement: Supplementary file 6 — Supplementary Figure 5 [file 41388_2023_2892_MOESM6_ESM.tif]
